# Selective Methylation of CpGs at Regulatory Binding Sites Controls *NNAT* Expression in Wilms Tumors

**DOI:** 10.1371/journal.pone.0067605

**Published:** 2013-06-25

**Authors:** Jochen Hubertus, Ferdinand Zitzmann, Franziska Trippel, Josef Müller-Höcker, Maximilian Stehr, Dietrich von Schweinitz, Roland Kappler

**Affiliations:** 1 Department of Pediatric Surgery, Research Laboratories, Ludwig-Maximilians-University, Munich, Germany; 2 Institute of Pathology, Ludwig-Maximilians-University, Munich, Germany; CNRS, France

## Abstract

Aberrant expression of imprinted genes, such as those coding for the insulin-like growth factor 2 (*IGF2*) and neuronatin (*NNAT*), is a characteristic of a variety of embryonic neoplasms, including Wilms tumor (WT). In case of *IGF2*, it is generally accepted that loss of imprinting in a differentially methylated region of the *IGF2/H19* locus results in biallelic expression and, thus, upregulation of the gene. In this study we examined methylation pattern at potential regulatory elements of the paternally expressed *NNAT* gene in a cohort of WT patients in order to further characterize the molecular mechanism causing overexpression of this regulatory gene. We demonstrate that transcriptional upregulation of *NNAT* in WT is grossly independent of the bladder cancer-associated protein (*BLCAP*) gene, an imprinted gene within the imprinted domain of the *NNAT* locus. However, expression of the *BLCAP* transcript isoform v2a formerly known to be selectively expressed from the paternal allele in brain was associated with high expression of *NNAT*. This contrasts the situation we found at the *IGF2/H19* locus, which shows high overexpression of *IGF2* and inversely correlated expression of the *H19* gene in WT. An analysis of DNA methylation in two potential regulatory regions of the *NNAT* locus by pyrosequencing revealed significant hypomethylation of the tumors compared to normal kidney tissue. Interestingly, the difference in DNA methylation was highest at CpGs that were observed within three putative binding sites of the CCCTC-binding factor CTCF. Most importantly, hypomethylation of both *NNAT* regulatory regions is significantly associated with the upregulation of *NNAT* expression and the *BLCAP*_v2a transcript. Our data indicate that the methylation status of a not-yet-described regulatory element within the *NNAT* locus that contains four potential CTCF binding sites determines the expression level of *NNAT* and the nearby located *BLCAP*_v2a transcript, thereby suggesting a functional role in the aberrant upregulation of *NNAT* in WT.

## Introduction

Genomic imprinting is the parent-of-origin silencing that results in monoallelic expression, with the associated allelic methylation at imprinting control (ICR) or differential methylated regions (DMR) being maintained irrespective of the transcriptional output [1]. These regions are characterized by cytosine-phosphate-guanine (CpG)-rich islands and contain binding sites for regulatory factors. High levels of methylation at these regions are suspected to prevent binding to regulatory elements such as promoters and insulators. The mechanism of genomic imprinting is known to silence developmental genes after the embryonic period [1]. Recent studies have shown that these imprinting patterns are aberrant in different neoplasms, especially in embryonal tumors [2]–[5].

Wilms tumor (WT), or nephroblastoma, accounts for 6% of all solid tumors in patients less than 15 years of age with an incidence of eight per one million children [6]. These malignant embryonal tumors are thought to develop from precursor cells at an early stage of metanephric differentiation [7]. Overexpression of the insulin-like growth factor 2 (*IGF2*) has been reported as a common characteristic in both WT and other embryonal tumors [8]–[10]. The *IGF2* gene is part of a cluster together with *H19*, a non-coding RNA gene, and the expression of both genes is regulated by a central DMR (*H19*DMR).To date, the *IGF2/H19* cluster and its regulation is the best-investigated imprinted region, and its role has been studied in many embryonal and adult cancers [4], [5], [9]. In somatic cells, *H19*DMR is methylated only on the paternal allele that expresses *IGF2* [11]. As a consequence, some regulatory elements, such as the CCCTC-binding factor (CTCF), are able to bind only on the maternal allele. CTCF is an 11-zinc-finger protein that acts as a cis-regulatory transcription factor with insulator activity [1]. It is generally accepted that loss of imprinting (LOI) at the *IGF2/H19* locus results in biallelic expression of the fetal growth factor *IGF2* and contributes to the development of embryonal tumors. However, overexpression of *IGF2* independent from LOI at the *H19*DMR has also been described for WT [12].

We have shown in a previous study that the aberrant methylation of *H19*DMR is associated with the overexpression of other imprinted genes, including neuronatin (*NNAT*) [2]. Those findings suggest a general overexpression of imprinted genes in WT by an as-yet-unknown superordinated mechanism. *NNAT* is a developmental gene involved in differentiation of neuronal tissues that may play a role in tumorigenesis [7]. Gene expression is regulated by a DMR spanning the first two exons [13]. *NNAT* is located in an imprinted domain within the intron of the bladder cancer-associated protein (*BLCAP*) gene [13]. Recent studies described a tissue and parent-of-origin specific expression of *BLCAP* and see the *NNAT* gene as a part of a *NNAT/BLCAP* imprinting cluster [14].

The aim of this study was to analyze the methylation pattern at regulatory elements of the *NNAT* gene in a cohort of WT patients and compare its influence on gene expression of *NNAT* and its neighbor *BLCAP*.

## Materials and Methods

### Patients

Forty-five WT specimens were investigated from patients undergoing surgical tumor resection in our department. The median age at time of surgery was 34.5 months (range 2 months to 17 years), with a sex ratio of 1∶1.5 (f:m). Thirty-seven patients (82%) underwent neoadjuvant chemotherapy according to the International Society of Pediatric Oncology (SIOP) protocol [15]. Eleven patients (24%) were found to have bilateral WT. The control group (n = 11) consisted of renal tissue from the healthy part of the resected specimen after tumor nephrectomy. The median age of the control group was 39 months (range 3 months to 5 years) with a sex ratio of 1∶2.7 (f:m). Histological classification of the samples was performed by a pathologist. The study was approved by the ethics committee of the Ludwig-Maximilians-University of Munich. Written consent was obtained from all parents.

### Real-time Reverse Transcription-PCR (RT-PCR)

Tri Reagent® was used for the isolation of total RNA from native samples. Total RNA and DNA were separated and subsequently purified using DNase and an RNeasy Mini Kit, respectively (Qiagen, Hilden, Germany). Reverse transcription of total RNA was performed using random hexamers (Roche Diagnostics, Penzberg, Germany) and SuperScript II reverse transcriptase (Invitrogen, Carlsbad, CA, USA). Intron-spanning primers were designed for the human genes *IGF2, H19, BLCAP,* and *NNAT* using Primer Express® v2.0 (Applied Biosystems, Foster City, CA, USA) based on the sequence information contained in the Ensembl Database. Primers were as follows (5′−>3′ orientation): *NNAT*: CGGCTGGTACATCTTCCGC, TGTCCCTGGAGGATTTCGAAA; *IGF2*: CCCGCTGGGCCAATCT, GAGTCTGGTTTTGATGCCACC; *H19*: CTCACCCACCGCAATTCATT, CGTGCCGGAGCTGCC; *BLCAP*: AACGGAAGCCTTGCACAATT, ATCGGAGCAGTGGTACAGGAA; *BLCAP*_v1a: GTGGCGAGCTGAGGTGGA, ATCGGAGCAGTGGTACAGGAA; *BLCAP*_v2a: TTTTCTGCTGGACAGGTCGTT, TGCTCTCTGGCTGTCAGCC.

Polymerase chain reaction (PCR) amplifications were carried out with 40 ng of cDNA, 500 nM forward and reverse primer and iTaq SYBR Green Supermix (Bio-Rad Laboratories, Hercules, CA, USA) in a final volume of 20 µl. The PCR reactions were run for 40 cycles consisting of 15 sec denaturation at 95°C, primer annealing for 15 sec at 55°C, and extension for 30 sec at 72°C on a Mastercycler Realplex^2^ cycler (Eppendorf, Hamburg, Germany). All PCR reactions were prepared in doublets and standardized to the reference gene TATA-Box-binding-Protein (*TBP*). The level of expression was calculated according to the mathematical model of Pfaffl et al. [16].

### Methylation Analysis using Pyrosequencing®

Double stranded DNA (dsDNA) was extracted from the native samples using standard procedures. Two micrograms of dsDNA were converted using the Epitec Bisulfite Kit® (Qiagen) according to the user manual. Pyrosequencing primers were designed with PyroMark Assay Design Software 2.0 and were as follows (5′−>3′ orientation):

*NNAT-*promoter: GGGGTAGGTATGGAAAGAGTAGA, AAACCCCCTCAAACTTACCTACAAC, GGTGGAGGGAGGGTATTTAA (sequencing primer); *NNAT*-CTCF: TGTTTGGGTTTTGTTTAGTAGAGAT, AATACTAAATACAAAACCCTATCCTTAC, GGTTTTGTTTAGTAGAGATTAT (sequencing primer); *BLCAP*-promoter: AGTTTTTGAGTTTTGGTTGTATGATGAA, AATCCCCTCATACATATACAACAA, GGGGTGGATTTTGAGTTA (sequencing primer); *H19*-DMR: GTATAGAGTTAGGGGGTTTTTGTATAGTA, CTCCCATAAATATCCTATTCCCAAATA, GGTTTTATAGTTTGGATGGT (sequencing primer). The DNA was amplified by PCR using 50 ng of dsDNA, 500 nM forward and reverse primer, and Maxima HotStart-Taq (Fermentas, Glen Burnie, USA) in a final volume of 15 µl. The PCR reactions were run for 45 cycles consisting of 20 sec denaturation at 95°C, primer annealing for 20 sec at 58.3°C, and extension for 35 sec at 72°C on a Mastercycler Realplex^2^ cycler (Eppendorf, Hamburg, Germany). PCR products were sequenced on a PyroMark™ Q24 instrument (Qiagen) using sequencing primer and ProMark™ Gold reagent as recommended by the manufacturer. Quantitative analysis of methylation was accomplished using the Pyro Q-CpG Software (Qiagen).

### Statistics

A statistical analysis was carried out with SPSS® v19.0. An explorative analysis was made without correcting p-values for multiple testing. The Mann-Whitney U-Test was used for comparison of gene expression data and methylation status. The relationship between two expression levels was measured by Spearman’s rank correlation.

## Results

### Gene Expression at the *IGF2*/*H19* and *NNAT*/*BLCAP* Imprinted Loci in Wilms Tumor

**Figure 1:**
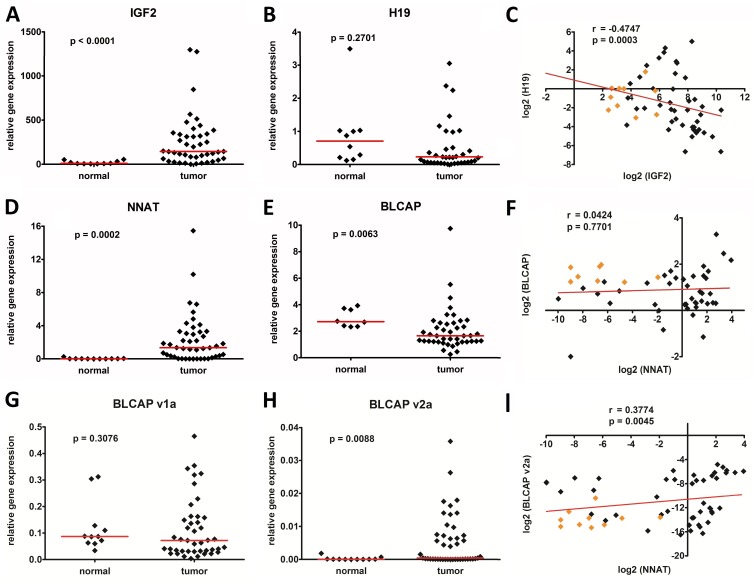
Relative expression of the genes A) IGF2, B) H19, D) NNAT E) BLCAP, G) BLCAP_v1a, and H) BLCAP_v2a in 45 Wilms tumors and 11 normal kidney tissues, as determined by real-time PCR. The dots represent relative candidate gene expression in relation to the house-keeping gene TBP. Median expression values are given as red horizontal lines. Association of C) IGF2 and H19, F) NNAT and BLCAP, as well as I) NNAT and BLCAP_v2a gene expression in log2 scale using Spearman’s rank correlation. Data from the tumor and normal kidney cases are depicted as black and orange diamonds, respectively.

First, we investigated the transcriptional activity of the genes *IGF2* and *H19* as well as *NNAT* and *BLCAP* in a cohort of 45 WT and normal kidney tissue. *IGF2* (p<0.0001) and *NNAT* (p = 0.0002) were significantly overexpressed, whereas expression of *BLCAP* (p = 0.0063) was significantly suppressed in tumors compared to healthy kidney tissue (Fig. 1a, d, e). The median *H19* expression level did not differ significantly from kidney (p = 0.2701) (Fig. 1b). As different *BLCAP* transcripts have been described [14], we further analyzed the expression level of the main v1a and v2a transcripts. Whereas the expression of *BLCAP*_v1a in tumors did not differ from kidney (p = 0.3076) (Fig. 1g), we found an overexpression of the *BLCAP*_v2a transcript in a subset of tumors (Fig. 1h). Of note, the *BLCAP*_v2a transcript was expressed at much lower levels than the *BLCAP*_v1a transcript.

Next, we correlated the expression level of the genes contained within the imprinted loci to each other using the Spearman`s rank correlation coefficient. We found that expression of *IGF2* is negatively associated with that of *H19* (p = 0.0003, r = −0.4747) (Fig. 1c), corroborating earlier findings that *IGF2* is negatively regulated by *H19* [2], [17]. In contrast, there was no correlation between *NNAT* and *BLCAP* (p = 0.7701, r = 0.0424) (Fig. 1f) as well as its v1a transcript (p = 0.1949, r = 0.1619) (not shown). Interestingly, there was a significant correlation between *NNAT* and *BLCAP_*v2a expression (p = 0.0045, r = 0.3774) (Fig. 1i) as well as *NNAT* and *IGF2* expression (p<0.001, r = 0.459) (not shown).

### DNA Methylation at the *IGF2*/*H19* and *NNAT*/*BLCAP* Imprinted Loci in Wilms Tumor

**Figure 2:**
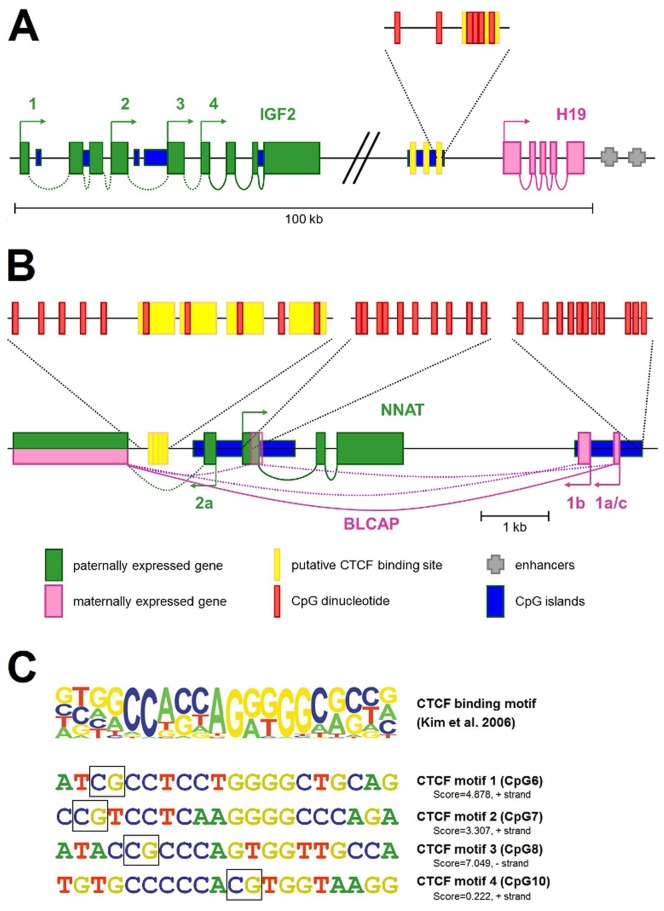
Schematic illustration of the A) IGF2/H19 and B) NNAT/BLCAP locus. Transcription starts are indicated by arrows and transcript variants are numbered. The CTCF binding sites (yellow bars) within the A) H19DMR and B) CpG-rich intergenic NNAT region as well as the NNAT and BLCAP promoter used for generating the DNA methylation assays are highlighted as magnifications indicating each CpG dinucleotide under investigation. C) Consensus sequence motif of CTCF-binding sites according to Kim et al. [28]. The height of each letter represents the relative frequency of occurrence of the nucleotide at each position. CTCF motifs 1–4 within the NNAT regulatory region with scores for similarity to the consensus, as determined by the JASPAR software (jaspar.genereg.net). CpGs under investigation are highlighted in black squares.

**Figure 3:**
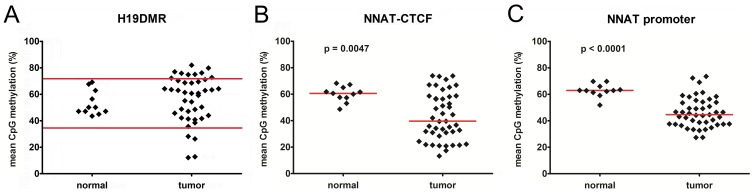
Mean DNA methylation levels at the A) H19DMR, B) the distal NNAT-CTCF binding site, and C) the NNAT promoter in normal kidney and tumor tissues. Red horizontal lines either depict two standard deviations above or below the mean of the normal controls (A) or the median DNA methylation values (B+C).

**Figure 4:**
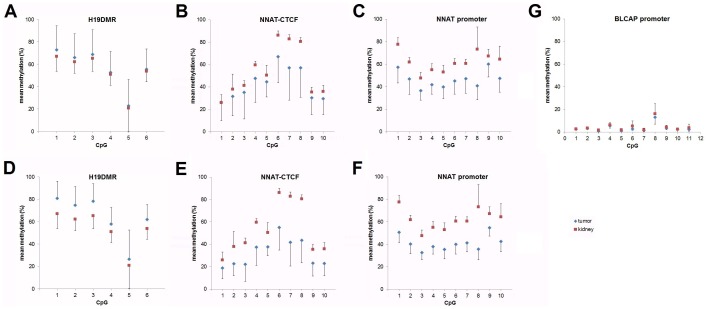
Mean DNA methylation levels delineated for each CpG at A) and D) the H19DMR, B) and E) the distal NNAT-CTCF binding site, C) and F) the NNAT promoter, and G) the BLCAP promoter in normal kidney (red squares) and tumor tissues (blue diamonds). A) to C) depict the DNA methylation levels for all tumor and normal kidney cases, whereas D) to F) depict only tumors with >10-fold mean expression of normal kidney for D) IGF2 (n = 22) and E) to F) NNAT (n = 33). Error bars represent the standard deviation of all tumor or kidney sample measurements.

To determine the methylation pattern at the *IGF2/H19* locus we established a pyrosequencing assay that analyzes six CpGs inside the *H19*DMR spanning one CTCF binding site (Fig. 2a). When calculating the mean methylation level across all six CpGs and defining hypo- and hypermethylation as a value of more than 2 standard deviations below and above the mean of the controls, we found all normal kidney samples and 32/45 tumors to display normal methylation values (Fig. 3a), thereby suggesting a monoallelic methylation pattern of the *H19*DMR in these cases. However, 9 of 45 tumors showed hypermethylation (mean methylation >71.8%), which implies biallelic methylation and, thus, loss of imprinting. Interestingly, we also detected four cases displaying hypomethylation (mean methylation <34.8%). By focusing at each individual CpG, we found that the mean methylation across all tumors was comparable to that of the kidney tissues (Fig. 4a), which is plausible because of tumor cases with normal, hyper- and hypo-methylation. Thus, we repeated the analysis by including only tumors with *IGF2* overexpression (n = 20 with >10-fold mean *IGF2* expression of kidney), assuming that these tumors should show the strongest methylation differences. Indeed, we detected significantly higher mean methylation levels in tumors compared to normal kidney samples for the six informative CpGs (Fig. 4d).

To analyze the methylation status of the *NNAT/BLCAP* locus, we first screened the genomic sequence around the *NNAT* and *BLCAP* coding sequence for potential imprinting control regions. We defined two regions of interest that are located inside of CpG islands and presumably lay within the *NNAT* and *BLCAP* promoter (Fig. 2b). A third region covers four potential CTCF binding sites 1.5 kb upstream of exon 1 (Fig. 2b+c) of the *NNAT* gene (Fig. 2b). By analyzing 10 CpGs of the *NNAT* CTCF binding site by pyrosequencing, we found a dramatic decrease in the overall methylation across all CpGs in the tumor samples (median 40%) versus normal kidney (median 60%) (Fig. 3b). By looking at the individual CpGs, we found considerable variation of methylation levels, ranging from 5%–100% (Fig. 4b). Generally, mean methylation levels were higher in normal kidneys than in tumor tissues, with the most significant differences in CpGs 6–8, which span the core region of the CTCF binding sites. This difference was even more pronounced when we included only tumors with *NNAT* overexpression (n = 33 with >10-fold mean *NNAT* expression of kidney) (Fig. 4e), thereby suggesting that hypermethylation of the potential CTCF binding site inside the *NNAT* CpG islands might play an important role in the physiological regulation of *NNAT*.

At the *NNAT* promoter region (Fig. 2b), we also found a strong reduction of the mean methylation level in tumor (median 45%) versus kidney tissues (median 63%) (Fig. 3c). In addition, the mean methylation rates at the individual CpGs differed considerably between normal kidney and tumor tissue, with significantly higher methylation levels in kidney (Fig. 4c). This difference remained nearly unchanged when including only the 33 tumors with *NNAT* overexpression (Fig. 4f). Interestingly, we detected a high correlation between the methylation of the *NNAT* CTCF binding site and the *NNAT* promoter (p<0.0001; *r* = 0.7234).

In contrast to the CTCF binding site and promoter region of the *NNAT* gene, the mean methylation at the *BLCAP* promoter was very low, ranging from 1 to 9%, and showed no differences between tumors and normal kidneys (Fig. 4g).

### Correlation of Gene Expression and DNA Methylation at the *IGF2/H19* and *NNAT/BLCAP* Imprinted loci in Wilms Tumor

To investigate the influence of the DNA methylation pattern at the *IGF2/H19* and *NNAT/BLCAP* imprinted loci on the transcriptional activity of the genes, we correlated methylation with expression using the Spearman-rank correlation.

**Figure 5:**
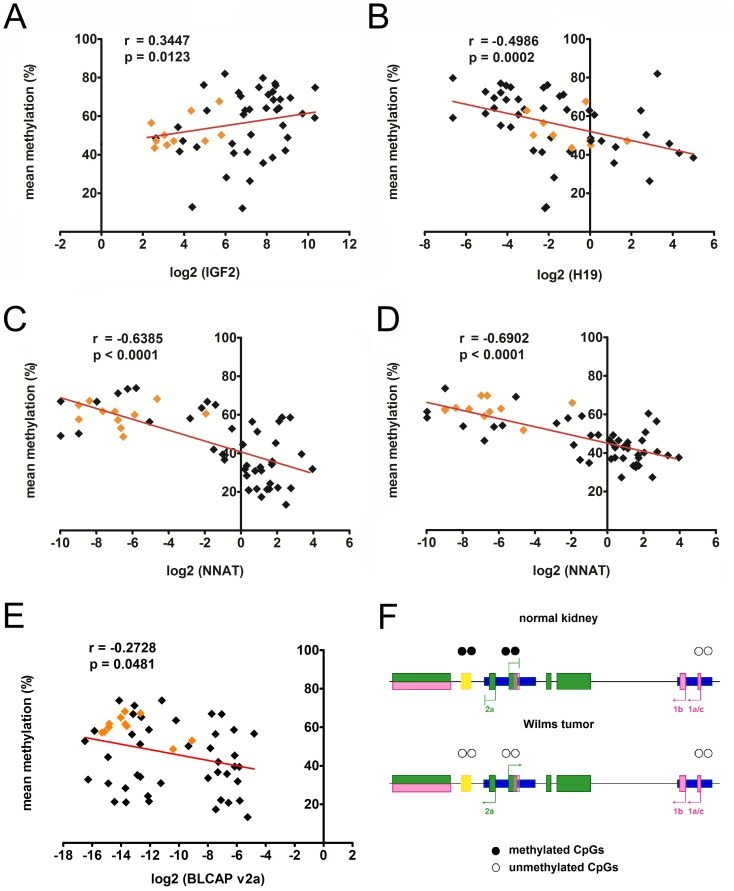
Association of gene expression and mean methylation of A) IGF2 and H19DMR, B) H19 and H19DMR, C) NNAT and NNAT-CTCF, D) NNAT and NNAT promoter, and E) BLCAP_v2a and NNAT-CTCF using Spearman’s rank correlation. The data from the tumor and normal kidney cases are depicted as black and orange diamonds, respectively. F) Model of the NNAT/BLCAP locus based on our data depicting suppression of BLCAP_v2a and NNAT in normal kidney due to methylated CpGs in the NNAT-CTCF site and NNAT promoter. Consequently, BLCAP_v2a and NNAT upregulation in Wilms tumors is caused by the demethylation of both regulatory regions. The BLCAP gene is expressed in both tumors and normal kidney due to unmethylted CpGs in its promoter.

At the *IGF2/H19* locus, we revealed a correlation between high methylation levels of the *H19*DMR and the overexpression of *IGF2* (p = 0.0123, r = 0.3447) (Fig. 5a). In sharp contrast, the second gene of interest at this locus, *H19*, was significantly suppressed by a high methylation level of the *H19*DMR (p<0.0002, r = −0.4986), as expected at this locus (Fig. 5b).

At the *NNAT/BLCAP* locus, high methylation levels at both the *NNAT* CTCF binding site (p<0.0001; *r* = −0.6385) and the *NNAT* promoter (p<0.0001; *r* = −0.6902) were associated with low *NNAT* expression levels (Fig. 5c+d). Although *NNAT* is located within the intron of *BLCAP*, mRNA expression of *BLCAP* including the *BLCAP*_v1a transcript was neither influenced by the methylation status of the *NNAT*-CTCF binding site nor of the *NNAT* promoter (data not shown). However, expression of the *BLCAP_*2a transcript was inversely correlated with methylation level at the *NNAT* CTCF binding site (p<0.0428; *r* = −0.2728) (Fig. 5e), but not the *NNAT* promoter (p<0.0580; *r* = −0.2621) (data not shown).

## Discussion

Overexpression of imprinted genes is a common phenomenon in several embryonal tumors, including WT. We previously speculated that this might be mediated by a yet unknown superordinated mechanism [2]. Here, we report on the comparative analysis of two imprinted genomic regions, the *IGF2/H19* and *NNAT/BLCAP* loci, in regard to gene expression and DNA methylation in a large collection of WT. Both loci were chosen due to the known overexpression of *IGF2* and *NNAT* in embryonal tumors [2], [18] and their key role in embryonic development [18], [19]. While *IGF2* is part of a gene cluster with the *H19*DMR being responsible for the expression of both *IGF2* and *H19* [20], the regulation of *NNAT* located within the intron of the gene *BLCAP* is less far understood [13]. Recent studies have postulated a tissue and transcript specific regulation of *NNAT* and *BLCAP* in a cluster, at least in the brain [14].

To date, the functional relevance of CTCF binding in regulating the expression of imprinted genes has been extensively studied at the *IGF2/H19* locus [9], [20], [21], but not at the *NNAT/BLCAP* locus. It is generally accepted that the methylation patterns of binding sites for regulatory proteins are responsible for the transcriptional regulation of many imprinted genes. CTCF is one of the factors that are involved in gene regulation, especially at the *H19*DMR and is well-studied at this locus [2], [11], [20]. By researching the Ensembl database, we detected a regulatory region of the *NNAT* locus that harbors four putative CTCF binding sites 1.5 kb upstream of the first exon of *NNAT* (Fig. 2b) and outside of its described promoter [13]. Thus, the aim of our study was to investigate the role of this CTCF binding region in the misregulation of the *NNAT* gene in WT and compare it to the respective site at the *IGF2/H19* locus.

In a first set of experiments we confirmed the well-known overexpression of *IGF2* in WT compared to kidney tissues (Fig. 1a and 1d) [2], [18], with the expected coordinate downregulation of *H19* (Fig. 1b) [2], [11], [17]. Moreover, we have shown hypermethylation of the CTCF binding site in 9 WT (Fig. 3a), which is in line with data described in earlier studies [2], [11], [17], [22]. As expected, there was a high correlation between hypermethylation and overexpression of *IGF2* (Fig. 5a) and a coordinate downregulation of *H19* (Fig. 5b). Using pyrosequencing, we were also able to analyze not only general methylation, but also the methylation of each single CpG in the region of interest, thereby revealing considerable differences with regard to methylation between each single CpG (Fig. 4a+d). However, these differences were only relevant for those tumors with a >10-fold mean expression of normal kidney (Fig. 4d). This could be explained by the inclusion of cases with hyper- and hypomethylation at *H19*DMR in our calculations (Fig. 1a). Nevertheless, the most significant differences in the degree of methylation were observed at CpGs 1–3 (Fig. 4d). Altogether, these data qualified our cohort of WT patients to be representative.

Next, we analyzed the transcriptional activity of *NNAT* and *BLCAP* in our samples. We thereby replicated the previously described overexpression of *NNAT* [2], [7], [23]. Aberrant transcriptional activity of *NNAT* has been reported in other tumors such as glioblastomas [24], neoplasms of eccrine, apocrine and sebaceous glands [25], malignant peripheral nerve sheath tumors [23], and others. Xu et al. described a correlation of *NNAT* overexpression and increased cell proliferation in glioblastomas associated with poorer outcome [24]. In contrast, *BLCAP* was generally suppressed in WT (Fig. 1e), when we simultaneously measured all transcript isoforms. However, by analyzing the expression of the two main *BLCAP* transcripts v1a and v2a that have their transcription starts either 5′ or 3′ of the *NNAT* gene we detected a more differentiated transcriptional activity. We found comparable expression levels of *BLCAP*_v1a in WT and normal kidneys, whereas the *BLCAP*_v2a transcript was overexpressed in a subset of tumors, even at very low levels (Fig. 1e, h). Analogous, we have seen a correlation of the *NNAT* and *BLCAP*_v2a expression (Fig. 1i). This may be explained by the postulated paternal expression of both, *NNAT* and *BLCAP*_v2a, while *BLCAP*_v1a seems to be maternally expressed [14]. Moreover, this finding suggests that *NNAT* and *BLCAP*_v2a might be regulated together by a shared regulatory element.

Indeed, we qualified a small region containing potential CTCF sites to be highly correlated with the expression of *NNAT* and *BLCAP*_v2a. The mean level of methylation of the potential CTCF binding sites and the promoter was lower in tumors compared to kidney tissues (Fig. 3b+c). Thus, our data suggest that the majority of WT exhibit hypomethylation of these two sites. This is contrary to findings in pituitary adenoma, which are characterized by a hypermethylation of adjacent CpG islands and a downregulation of *NNAT* [26]. This might be explained by the finding that the pituitary gland is the only adult tissue with high transcriptional activity of *NNAT* [26]. From the mechanistically point of view, our data are in accordance with Revill et al. showing a strong association of hypermethylation and gene silencing for the promoter and, even more interestingly, with the newly described potential CTCF binding site. While all of the promoter CpGs had a lower mean level of methylation in tumors (Fig. 4c+f), methylation especially of CpGs 6–8 containing three of the four potential CTCF binding sites, considerably differed from kidney tissue (Fig. 4b+e). As seen at *H19*DMR (Fig. 4a+d), these differences are intensified when we look at tumors with relevant increased expression levels (Fig. 4e). However, since the methylation status of both the potential CTCF binding site and the promoter are correlated with the level of expression (Fig. 5c–d), we cannot discriminate the influence of each individual regulatory element on gene regulation. In addition, we found no methylation of the *BLCAP* promoter, which is in line with data from fetal brain and other tissues [13], thereby explaining the general expression of *BLCAP* in both the tumors and the normal kidney tissues.

Finally, because CTCF functions as an insulator [20], [22], [27] and the *NNAT* CTCF binding site is placed inside a *BLCAP* intron, a potential influence on *BLCAP* expression can be assumed. However, there was no correlation of the total *BLCAP* and *BLCAP_*v1a expression with methylation at the potential *NNAT* CTCF binding site. However, the *BLCAP_*v2a transcript was expressed in tumors only and was correlated with the *NNAT* expression as well as the methylation of the *NNAT* CTCF binding site. These findings corroborate the results of Schulz et al. [14], which described a transcript and tissue specific imprinting of the *NNAT/BLCAP* locus. Nevertheless, the reactivation of *NNAT* and other imprinted genes may be attributed to genome-wide altered methylation of regulatory binding sites. The question of whether the potential *NNAT* CTCF binding site in WT has never been methylated and mirrors the embryonic state rather than being actively demethylated during tumorigenesis cannot be answered to date.

In conclusion, our data suggest that, in contrast to the *IGF2/H19* locus, hypomethylation of regulatory elements are associated with *NNAT* overexpression. We have shown that differences in methylation are most prominent at a few CpGs covering potential CTCF binding sites, whereas differential methylation of the *NNAT* promoter region is evident over a large proportion. This might lead to the concomitant overexpression of *NNAT* and *BLCAP_*v2a transcripts in WT, while leaving the general expression of *BLCAP* unaffected. Thus, the process of aberrant methylation in WT seems to be a targeted and site-specific process that impacts selected regulatory sites rather than a general ongoing mechanism acting throughout the genome. This suggests that methylating enzymes may be reliant on motifs in the DNA.
